# Effect of Garlic Straw with Silage Corn Stalks on Hu Sheep Rumen Fermentation and Microbial Community In Vitro

**DOI:** 10.3390/metabo13121201

**Published:** 2023-12-17

**Authors:** Yangyang Shen, Jianli Zhang, Hongbing Gui, Huili Wang, Yinxia Li, Jun Zhang, Shaoxian Cao, Jifeng Zhong, Yong Qian, Chunhua Meng

**Affiliations:** 1 Institute of Animal Science, Jiangsu Academy of Agriculture Sciences, Nanjing 210014, China; 2019205005@njau.edu.cn (Y.S.); zhangjianli79@163.com (J.Z.); guihongbing1715225@163.com (H.G.); wanghuili318@163.com (H.W.); liyxmh@jaas.ac.cn (Y.L.); applepuding@163.com (J.Z.); sxcao@jaas.ac.cn (S.C.); zhongjifeng64@sina.cn (J.Z.); 2 Key Laboratory of Crop and Animal Integrated Farming, Ministry of Agriculture and Rural Affairs, Nanjing 210014, China; 3 Jiangsu Key Laboratory for Food Quality and Safety-State Key Laboratory Cultivation Base of Ministry of Science and Technology, Nanjing 210014, China; 4 School of Animal Husbandry and Veterinary Medicine, Jiangsu Vocational College of Agriculture and Forestry, Jurong 212400, China

**Keywords:** garlic straw, Hu sheep digestibility, ruminal fermentation, volatile fatty acids, microbiota

## Abstract

Garlic, an important economic crop, provides nutrient-rich straw. When appropriately balanced with silage corn stalks, it is a high-quality forage resource. However, studies on the impact of garlic straw with silage corn stalks on Hu sheep’s digestive metabolism and rumen microbiota are scarce. In this study, different addition ratios of garlic straw and silage corn stalks were utilized for in vitro experiments. We designed six experimental groups (CON, G0, G20, G40, G60, G80, and G100) based on varying ratios of garlic straw to silage corn stalks. Rumen microbiota was analyzed through 16S rRNA sequencing. Nutrient composition analysis indicated that garlic straw’s relative feeding value (RFV) closely resembled that of silage corn stalks. After 24 h of fermentation, dry matter digestibility and in vitro gas production significantly increased, reaching peak values at a 60% addition ratio. Furthermore, volatile fatty acids (VFAs) such as acetic, propionic, and butyric acid exhibited elevated contents, with the highest yields observed at 60% inclusion. At the genus level, *Prevotella*, *Rikenellaceae RC9* gut group, and *Succiniclasticum* were identified as the dominant bacterial groups. The gas production test showed a significant decrease in the G80 group compared to others. Microbial analysis revealed a higher abundance of *Prevotella* in G80 compared to G20, offering valuable insights for reducing greenhouse gas emissions from ruminant animals. Finally, this study predicted the impact of garlic straw with silage corn stalks’ addition on Hu sheep’s metabolic pathways and biological functions of the rumen microbiota. This research highlights the potential for effectively utilizing garlic straw as a feed resource for Hu sheep and proposes a rational proportion for combining garlic straw with silage corn stalks.

## 1. Introduction

Chinese garlic production and planting area constitute a significant portion of the global market, with 80.86% and 58.43% shares, respectively, making China the world’s leading exporter and consumer of garlic [1]. However, the remaining garlic straw poses a challenge for composting and field reintegration due to its bactericidal properties after each harvest [2]. In certain regions, it is either burnt for soil improvement or used as feed [3]. Utilizing garlic straw as feed addresses its environmental impact and contributes to the availability of raw materials for ruminant roughage, thereby enhancing farmers’ income. With the recent expansion of large-scale sheep farming in China, characterized by intensive house feeding, there has been a surge in the prices of roughage, corn, and other feed raw materials, accounting for approximately 70% of breeding costs [4]. Consequently, finding alternative feed sources to offset some ingredients is crucial for cost-effective sheep production.

Garlic straw, the above-ground component of garlic post-harvest, boasts a nutritional profile akin to garlic. It contains vital active compounds such as allicin and superoxide dismutase, endowing it with antibacterial, anti-inflammatory, immune-boosting, cardiovascular-protective, and anti-tumor properties [5]. When employed as a feed additive, it offers the advantages of growth promotion, enhanced livestock product quality, eco-friendliness, and a potential partial replacement for antibiotics. The crude protein content in garlic straw parallels that of alfalfa hay, with higher levels of neutral and acid detergent fiber. Extensive utilization in enhancing the growth of various animals like chicken [6], sheep [7], rabbits [8], and buffalo [9] underscores its remarkable applicative value. However, due to its inherent antibacterial properties, judicious consideration of the inclusion level is imperative.

The mammalian gut microbiota is essential in shaping many of the host’s functional properties, with the rumen of sheep being the first site of interaction between the host and ingested nutrients [10]. Rumen microbiota includes bacteria, archaea, fungi, and protozoa. Its ability to accurately sense and respond to fluctuations in dietary nutrient levels is essential for the metabolic balance of ruminants [11]. However, the effect of garlic straw with silage corn stalks on rumen fermentation needs further study.

Given that livestock feed constitutes a substantial portion of animal agriculture production costs, enhancing feed efficiency is paramount, especially in escalating feed expenses or declining livestock values. Elevating feed efficiency can curtail feed consumption while upholding animal performance levels [12]. Although investigations into feed efficiency and rumen microbial profiles of sheep have been limited thus far, the advent of advanced sequencing technology has led to a surge in research in this domain [13]. This study leverages chemical analysis, in vitro gas production, and high-throughput 16S sequencing techniques to appraise the nutritional worth of garlic straw, along with its impact on rumen fermentation parameters and microbiota in Hu sheep. This research aims to provide a theoretical hypothesis incorporating garlic straw into livestock feed, ultimately contributing to an improved feed efficiency and more sustainable animal agriculture practices.

## 2. Materials and Methods

### 2.1. Determination of Nutritional Composition of Garlic Straw

Dry matter was determined according to AOAC standard procedure. The following methods were employed to determine specific nutrients/elements. Crude fat (EE): the determination of crude fat content was conducted following the AOAC official method (AOAC 954.02). Calcium (Ca) and phosphorus (P): the calcium and phosphorus content in garlic straw were determined using AOAC official methods (AOAC 968.08 for calcium and AOAC 965.17 for phosphorus). Acid detergent fiber (ADF): the acid detergent fiber content was determined using the AOAC official method (AOAC 973.18). These methods are widely accepted in the field and ensure the accuracy and reliability of the nutritional composition data. The nitrogen content of the feed was determined by the Kjeldahl method, and the neutral detergent fiber NDF was determined according to Van Soest [7].

### 2.2. Feed Ingredients Collection

Garlic straw and silage corn stalks were collected from Shanxian City (Shandong Province, China), dried at 70 °C, ground, and sifted 1mm for storage. Nutritional composition is shown in Table 1.

### 2.3. Animal Feeding Management and Rumen Fluid Collection

Rumen fluid was collected from 4 healthy Hu sheep weighing 50~75 kg and fitted with permanent rumen fistulas, maintaining a roughage-to-concentrate ratio of 1:1 (at Liuhe Animal Science Base of Jiangsu Agricultural Science Academy). Complied guidelines for animal care were carefully observed to ensure all animals were treated compassionately and with regard for all deviation of suffering. Moreover, all experiments were carried out in strict accordance with the guidelines and rules. According to the National Scientific Research Council meat sheep feeding standards [14], the growth nutritional requirements of medium-fattening lambs were formulated (Table 2) to meet the development of Hu sheep. The rumen fluid was collected in a thermos bottle filled with CO_2_, and the temperature was 38–40 °C. The rumen fluid was returned to the laboratory and filtered with four layers of gauze.

### 2.4. In Vitro Fermentation

In vitro rumen fermentation was performed according to Menke et al. (1979) method [15]. The preparation of artificial rumen fluid is shown in Table 3. Add 380.45 mL distilled water, 0.1 mL A liquid, 190.23 mL B liquid, 190.23 mL C liquid, 0.95 mL Azurin solution, mix well, and add the reducing agent solution (38.05 mL) before mixing with the rumen fluid and place it in a 39 °C water bath. Then, introduce carbon dioxide to maintain an anaerobic environment until the mixture becomes lighter in color. Mix the artificial rumen fluid with the filtered rumen fluid at a ratio of 2:1, and the process continues to pass CO_2_. After the fermentation substrate is mixed according to the ratio of garlic straw to silage corn stalks 0:100, 20:80, 40:60, 60:40, 80:20, and 100:0, set up four replicates in each group. Accurately weigh 0.4 g fermentation substrate for each repeat in the bottle, quickly add 50 mL fermentation liquid to the bottle, and pass into CO_2_. The fermentation bottle was incubated in a constant temperature (39 °C) tank for 24 h, and the gas production was measured. After fermentation for 24 h, take out the bottle and measure the pH value with a pH meter. The pH value was measured with an EL20 acidity meter and gas production was measured with a gas meter (CRP500, ChenRui, Technology, Shenzhen, China).

### 2.5. Dry Matter Digestibility, Amino Acid Concentration, and Fermentation Parameters

Substrate residues post-fermentation were gathered in nylon bags and subjected to 24 h drying in an oven at 60 °C to ascertain in vitro dry matter digestibility. The fermentation substrate dry matter was determined by oven drying method. Dry matter digestibility/% = (M1 − M2/M1) × 100 (where the sample weight is M1, g, and the residue weight after in vitro digestion is M2, g). VFAs were assessed through gas chromatography (Agilent 6890N, Agilent Technologies Inc., Nanjing, China) using a DB-FFAP capillary column (30 m length, 0.25 mm diameter, 0.25 μm film thickness) with crotonic acid as the internal standard. Initially, the rumen fluid sample was extracted at −20 °C, fully thawed at 4 °C, and thoroughly mixed. Subsequently, 1.2 g of acidifying adsorbent, 1.5 mL chloroform solution of crotonate (5 mmoL/L), and 0.5 mL of rumen fluid samples were introduced into a 10 mL transparent centrifuge tube. Centrifuge at 4 °C 8000 r/min for 15 min to remove feed particles and impurities. The tube was promptly sealed, and 200 μL supernatant was extracted to analyze various volatile fatty acids. The determination conditions were as follows: carrier gas N2, flow rate 50mL/min, pressure 15.67 psi, flow rate 2.8 mL/min, constant flow; column box temperature ranged from 140 ℃ (4 min) to 240 ℃ (2 min), with an injection rate of 50 ℃/min, an injection ratio of 30:1, an injection volume of 1 μL, and an injection temperature of 185 ℃.

### 2.6. DNA Extraction and Sequencing

Microbial DNA was extracted from rumen fluid samples using HiPure Soil DNA Kits (or HiPure Stool DNA Kits) from Magen (Guangzhou, China), following the manufacturer’s protocols. The purity and concentration of DNA were assessed using a NanoDrop2000 UV-VIS spectrophotometer from Thermo Scientific (Wilmington, DE, USA). DNA integrity was verified through 1% agarose gel electrophoresis. Amplification was performed using primers targeting the 16S rRNA (V3-V4) region with the following sequences: 341F: CCTACGGGNGGCWGCAG and 806R: GGACTACHVGGGTATCTAAT. The PCR reagents used were TransGen High-Fidelity PCR SuperMix from TransGen Biotech (Beijing, China), with a reaction volume of 50 μL, forward and reverse primers at a concentration of 0.2 μM, and template DNA at 5 ng. Subsequently, PCR products were gel-purified quantified using a QuantiFluorTM fluorescence spectrophotometer, and the purified amplicons were equimolarly pooled, ligated with sequencing adapters, and prepared as sequencing libraries for Illumina PE150 sequencing on Hiseq X Ten platform (Guangzhou Kidio Biotechnology Co., Ltd., Guangzhou, China). Due to the large number of low-quality data or non-biological data (such as chimeras) generated by PCR errors and sequencing errors, in order to ensure statistical reliability and biological validity of follow-up analysis, we used DADA2 software to conduct strict quality control and obtain clean tags for follow-up analysis. Reads were filtered and calibrated using DADA2, and non-redundant reads and corresponding abundance information were output. Then, reads were spliced into tags, and chimera tags were removed to obtain tag sequences and abundance information, namely ASV sequences and ASV abundance information, for subsequent analysis.

### 2.7. Bioinformatic and Statistical Analysis

The bioinformatics analyses were conducted using R (version 4.3.1) and the Gene De novo platform (https://www.omicshare.com/, accessed on 1 July 2023). α- and β-diversity parameters were computed from normalized data with QIIME version 1.8.0. Canonical correspondence analysis (CCA) or redundancy analysis (RDA) was performed using the vegan package in R software version 2.15.3. The choice between CCA and RDA was based on the lengths of the first axis gradient in the data analysis results. If the lengths exceeded 4.0, CCA was chosen; RDA and CCA were used if they fell between 3.0 and 4.0. If the lengths were less than 3.0, RDA was utilized.

When evaluating dose–response, regression analysis is the most recommended method, instead of mean comparison tests. So, linear and quadratic effects were tested for gas production and fermentation solution digestibility, but the result was not significant here (*p* > 0.05). All statistical analyses were performed using SPSS software (version 20.0 for Windows) and assessed by one-way ANOVA and Student–Newman–Keuls’ post hoc analysis after testing for homogeneity of variance. Data are expressed as the mean ± SE. Differences with a *p*-value < 0.05 were considered significant.

## 3. Results

### 3.1. In Vitro Fermentation Responses to Varying Garlic Straw Proportions

Compared to the control (CON) group, the introduction of garlic straw led to an augmented gas production (Table 4). As the inclusion ratio ascended from 0% to 60%, gas production experienced a concurrent increase. Upon reaching the maximum gas production at 60%, further elevating the inclusion ratio reduced gas production, which remained significantly different from the CON group (*p* < 0.001). The pH value exhibited a notable decrease following the addition of garlic straw when compared to CON. However, by incorporating a small amount (20%) of garlic straw, the pH remained increased. When the inclusion ratio exceeded 60%, the pH value exhibited a significant alteration compared to the CON group (*p* < 0.05). In comparison to the G0 group, in vitro dry matter digestibility (IVDMD) showed significant disparities (*p* < 0.001). Dry matter digestibility experienced a decline at 20% and reached its zenith at 60%, and further increments did not yield significant changes.

### 3.2. Effects of Different Proportions of Garlic Straw on VFA In Vitro Fermentation

We investigated the influence of varying proportions of garlic straw with silage corn stalks on VFAs’ in vitro fermentation (Table 5). The table displays concentrations of volatile fatty acids (VFAs) in distinct treatment groups with varying ratios of garlic straw and silage corn stalks. Acetic acid, a key VFA, reached its highest concentration in the G60 group (25.30 mmol/L) and its lowest in the G40 group (20.64 mmol/L). For propionic acid, the highest concentration occurred in the G60 group (11.58 mmol/L), while the lowest was in the CON group (7.72 mmol/L). Butyric acid concentrations mirrored this pattern, with the G60 group (5.67 mmol/L) registering the highest and the G40 group (4.82 mmol/L) the lowest concentrations. Total VFA concentrations peaked in the G60 group (42.55 mmol/L) and dipped in the G40 group (34.12 mmol/L). This analysis provides nuanced insights into the influence of garlic straw and silage corn stalks ratios on VFA concentrations, shedding light on their impact on rumen fermentation dynamics in vitro.

### 3.3. Analysis of Rumen Flora Composition

**Figure 1:**
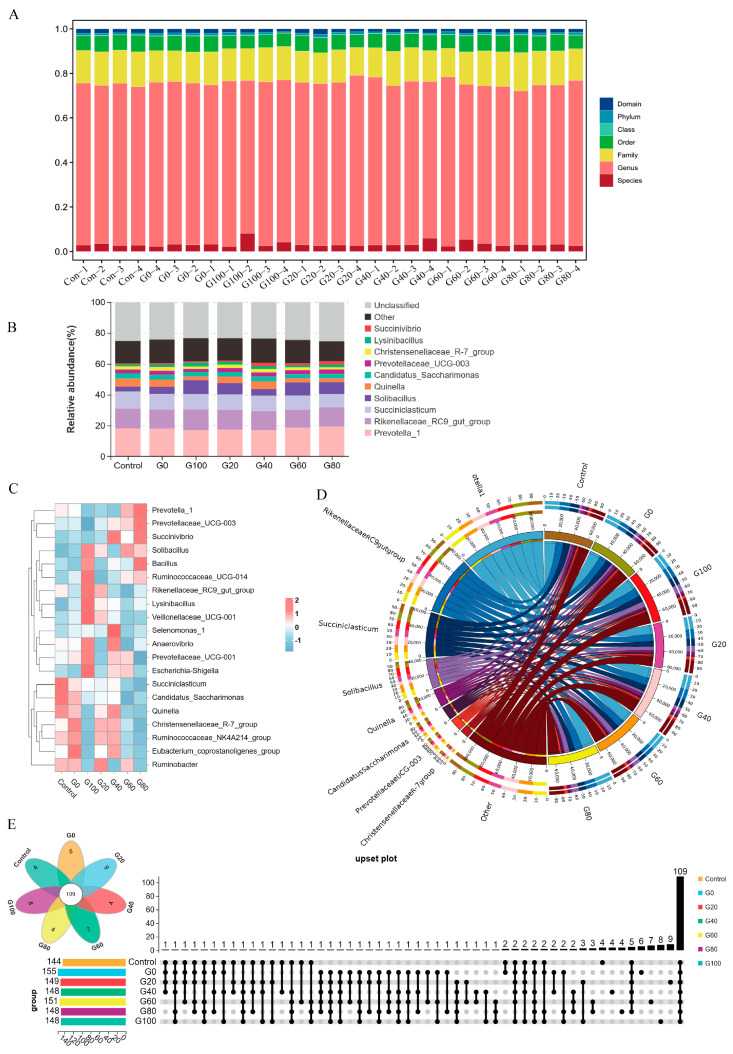
Different groups of rumen microbiota composition in Hu sheep. (**A**) Bar chart showing the sequence composition at various taxonomic levels (percentage). Based on the species annotation information of ASVs, the number of tag sequences in each sample at various taxonomic levels (domain, phylum, class, order, family, genus, species) was counted and plotted in a bar chart. (**B**) Relative abundance of genus species, visually displaying the variation in species abundance at different taxonomic levels among different samples. The top 10 species with the highest average abundance in all samples are detailed, while the remaining species are categorized into the “Other” category. Tags that cannot be annotated at this level are classified into the “Unclassified” category. (**C**) Genus-level species’ classification heatmap. The species selected for heatmap analysis must have a relative abundance (species tag count/total tag count) of at least 0.1% in at least one sample. (**D**) Taxa Circos plot at the genus level, including all species with a top 10 abundance ranking and tag counts greater than 2000 in all samples/groups. (**E**) Genus-level species’ Venn diagram.

The majority of ASVs were predominantly classified at the genus and family levels, with a discernible yet comparatively lower proportion at the species level, as depicted in Figure 1A. The subsequent examination of genus-level species’ composition, illustrated through a stacked bar plot (Figure 1B,C), highlighted the prevalence of taxa such as *Prevotella*, *Rikenellaceae RC9* gut group, and *Succiniclasticum* across diverse samples. A Circos plot was employed to visualize the distribution patterns and correlations of the top 10 abundant taxa at the genus level (with over 2000 tags) in all grouped samples (Figure 1D). This visualization offered a nuanced understanding of the relationships among the most prevalent genera. Furthermore, an indicator species analysis was conducted to identify taxa with a discriminative value between different groups. The Venn diagram vividly demonstrated 109 species’ match among the representative groups at the genus level (Figure 1E). These results collectively provide a comprehensive view of the rumen flora composition, emphasizing the prevalence of specific taxa and their potential discriminative value across different experimental groups.

### 3.4. Alpha Diversity

**Figure 2:**
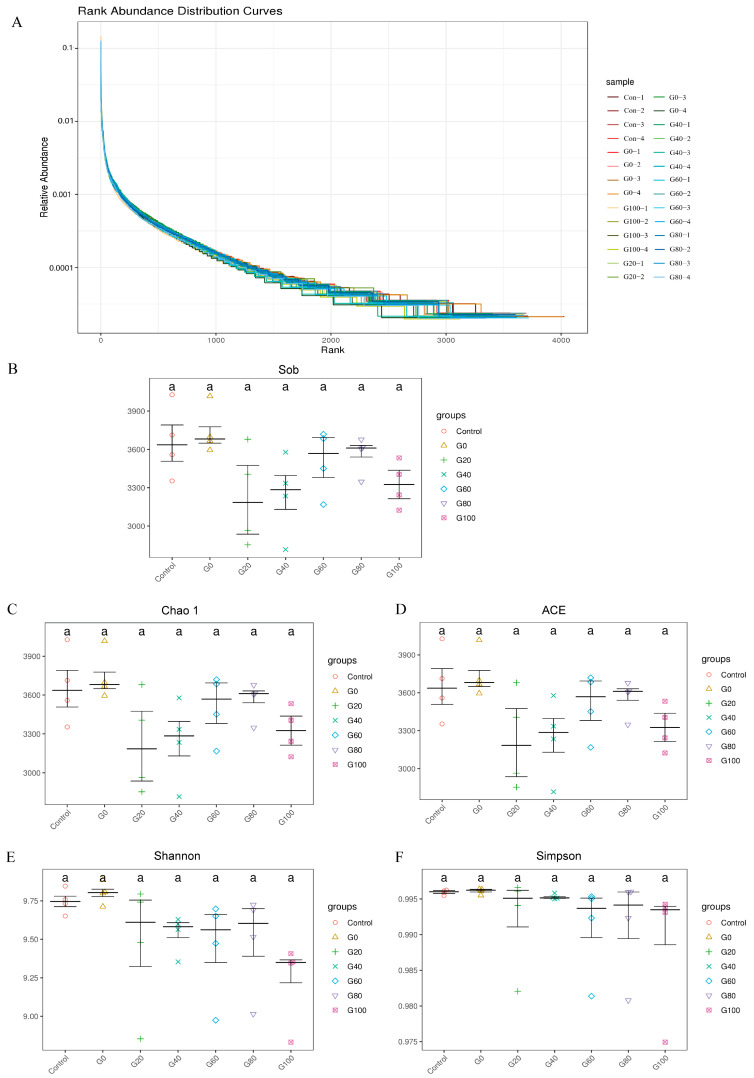
Alpha diversity analysis diagram for each group. (**A**) Rank abundance curves of samples in each group. The ASVs in the samples are sorted by relative abundance (or the number of sequences contained) from largest to smallest, resulting in corresponding ranking numbers. These ranking numbers are used as the horizontal axis, while the relative abundance (or the relative percentage of sequence number in the ASVs at this level) is used as the vertical axis. The points are connected by lines to create rank abundance curves. In the horizontal direction, the width of the curve reflects the abundance of classification; the smoothness of the curve in the vertical direction reflects the evenness of species distribution in the sample. Box plots based on Tukey’s HDS test results are plotted to display the degree of significant differences in diversity indices between pairwise groups in multi-group comparisons. (**B**) Sob represents the number of ASVs and observed species detected by sequencing. (**C**) Chao1 and (**D**) ACE represent the species’ richness information of the samples. (**E**) Shannon and (**F**) Simpson comprehensively reflect the richness and evenness of species. In peer data, different lowercase letters of shoulder labels indicate a significant difference (*p* < 0.05), and the same or no letters indicate no significant difference (*p* > 0.05).

The rank abundance curve visually represents the taxonomic richness and evenness within the samples (Figure 2A). We observed that the curves for each group were widespread along the horizontal axis, with smooth curves in the vertical direction, indicating a relatively even distribution of species. Additionally, α diversity analysis revealed high values of diversity indices, including Sob (Figure 2B), Chao1 (Figure 2C), ACE (Figure 2D), Shannon (Figure 2E), and Simpson (Figure 2F), among the rumen fluid samples in each group. However, the differences between groups were not statistically significant (*p* > 0.05).

### 3.5. Microbial Analysis of Rumen Bacterial Flora Differences between Groups

**Figure 3:**
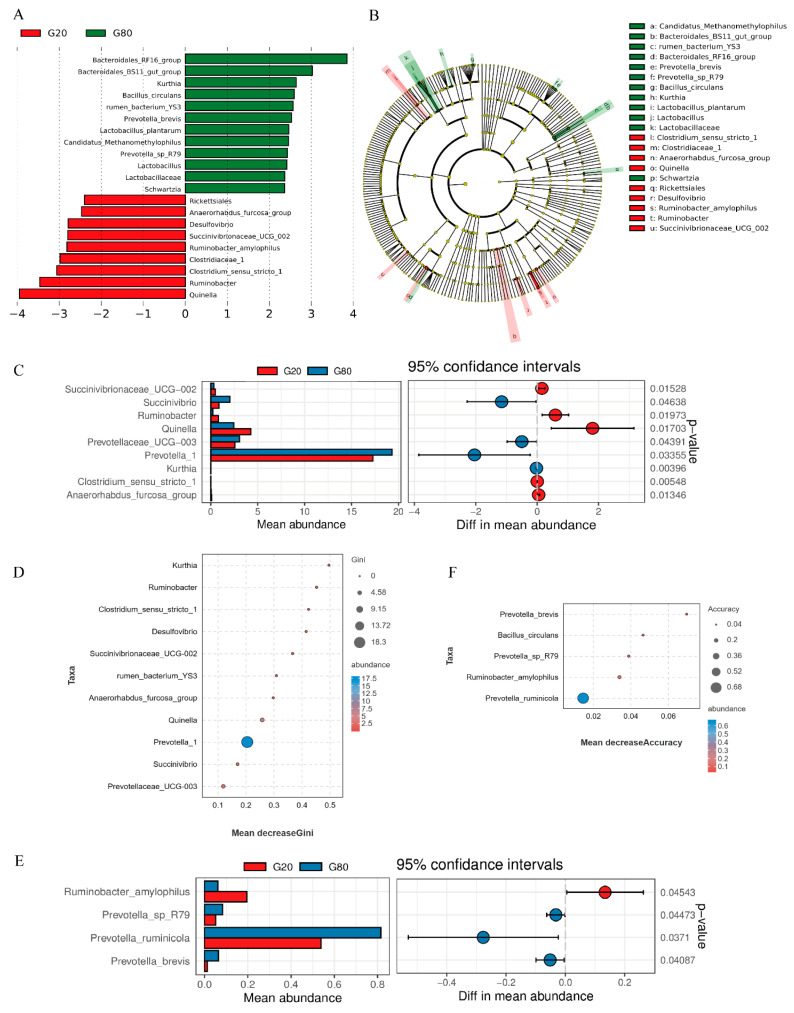
Microbial analysis of rumen bacterial flora differences between groups. (**A**) The bar graph represents significantly different bacterial taxa identified by the linear discriminant analysis effect size (LEfSe). The length of the bars represents the effect size of the differential species (i.e., the LDA Score). (**B**) Evolutionary branching diagram obtained by mapping the differences to the known hierarchical structure (right graph). In the evolutionary branching diagram, the concentric circles radiating from the center represent the classification levels from phylum to genus (or species). Each small circle at different classification levels represents a classification at that level, and the diameter of the small circle is proportional to the relative abundance. Coloring principle: species with no significant difference are uniformly colored yellow. (**C**) Bar graph showing genus-level differences in *t*-test. (**D**) Scatter plot showing genus-level differences in random forest contribution. (**E**) Bar graph showing genus-level differences in *t*-test. (**F**) Scatter plot showing species-level random forest contribution.

In this study, we observed a significant decrease in gas production and in vitro dry matter digestibility in the G80 group. However, there was no significant difference in the production of volatile fatty acids during in vitro rumen fermentation. To explain this phenomenon, we chose the G20 group as a comparison and conducted LEFse analysis to assess the impact and evolutionary divergence of microbial differences between the groups, aiming to identify specific microbial taxa in the G80 group (Figure 3A,B). The results revealed that 21 bacteria at different taxonomic levels had LDA values greater than 2, with 9 in the G20 group and 12 in the G80 group. To further compare the microbial differences between the G20 and G80 groups, we conducted Welch’s *t*-test and random forest analysis separately for both groups. At the genus level, we found that compared to the G20 group, the G80 group showed a significant increase (*p* < 0.05) in *Succinivibrio*, *Prevotellaceae_ucg-003*, and *Prevotella_1*, while *Succinivibrionaceae*, *Ruminobacter*, and *Quinella* showed a significant decrease (*p* < 0.05) (Figure 3C). At the species level, we observed a significant increase (*p* < 0.05) in *Prevotella* and a significant decrease (*p* < 0.05) in *Ruminobacter* in the G80 group compared to the G20 group (Figure 3E). Random forest analysis also indicated that *Succinivibrionaceae* and *Ruminobacter* significantly contributed to the differences between the two groups (Figure 3D,F).

### 3.6. Effect of Garlic Straw with Silage Corn Stalks on the Functional Pathway of Rumen Bacteria in Hu Sheep

**Figure 4:**
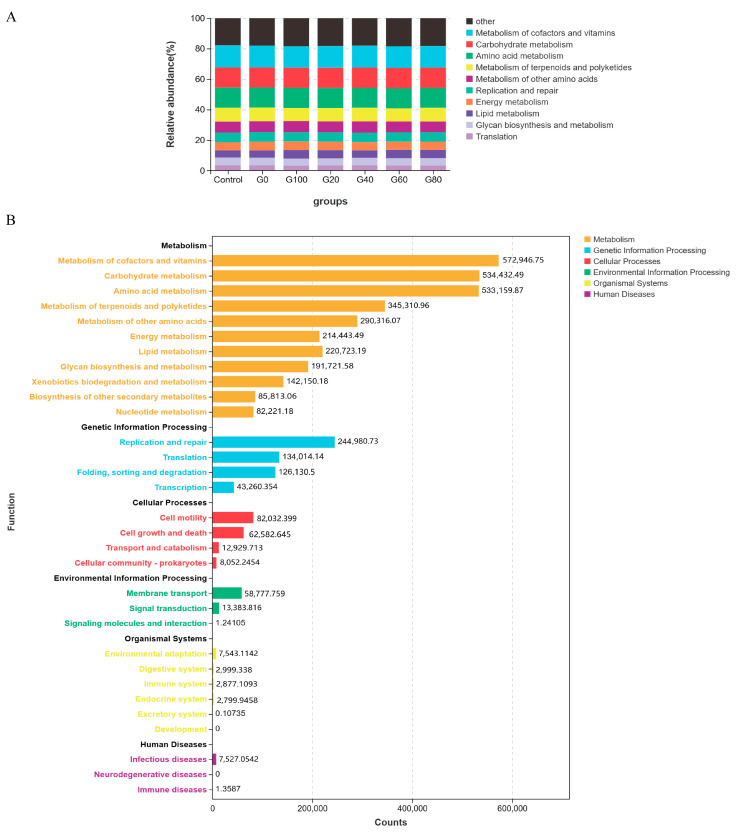
Concentration map of rumen bacteria predicting functional pathways in Hu sheep. (**A**) Stacked graph of predicted KEGG pathway functional annotations for each group. (**B**) Functional classification of biological metabolic pathways at levels A and B. Based on the species’ annotation and abundance information of ASVs, PICRUSt2 software was used for functional annotation of KEGG pathways of bacteria/archaea (16S), and the abundance information of each pathway was calculated. The bar graph displays the abundance of different functional categories (count values).

Using PICRUSt2-based functional prediction and metabolic capacity analysis, we predicted the impact of garlic straw with silage corn stalks’ addition on Hu sheep’s operating characteristics of the rumen microbial community. We found no significant differences between the control group and the treatment group. At the first level, the primary enrichment was observed in the metabolic pathways related to the metabolism of cofactors and vitamins, carbohydrate metabolism, amino acid metabolism, and metabolism of terpenoids and polyketides (Figure 4A). In detail, enrichment was observed in the metabolism related to the metabolism of cofactors and vitamins, amino acid metabolism, and carbohydrate metabolism. Genetic information processing is mainly associated with replication, repair, and translation. Cellular processes are primarily related to cell motility. Environmental information processing is explicitly linked to membrane transport (Figure 4B).

## 4. Discussion

### 4.1. Effects of Different Addition Ratios on In Vitro Gas Production, pH, and Dry Matter Digestibility

Numerous studies have demonstrated the direct influence of diet composition on rumen fermentation indices [13]. Our findings corroborate this relationship. The incorporation of garlic straw impacts various indicators, with gas production as a pivotal measure of rumen fermentation activity, providing insight into the action of rumen microorganisms. While previous research indicated that substances derived from garlic, such as garlic leaf [14], garlic powder [15,16,17], garlic oil [18], allicin [19], and other extracts were able to reduce methane production, our study yielded consistent results in the G80 group. Specifically, the addition of silage garlic straw led to an increase in the rumen gas production in Hu sheep. This outcome may be attributed to variations in the concentration of active compounds and processing techniques. Further research is required to determine if garlic straw directly impacts CH_4_ production. Moreover, we observed that gas production exhibited no significant change when the proportion was below 60%. However, gas production decreased when the ratio exceeded 60%, suggesting a degree of ruminal tolerance to garlic straw.

Maintaining an appropriate pH level is fundamental to optimal rumen activity. An excessively low pH can result in acidosis, while unduly high pH can inhibit the activities of crucial bacteria, thereby affecting rumen fermentation [20,21,22]. Including small amounts of garlic straw (0%, 20%, 40%) did not lead to significant changes in pH. Furthermore, pH levels stabilized when the proportion exceeded 60%, indicating minimal impact on ruminal fermentation [21]. Garlic straw exhibits a protein content akin to alfalfa, with higher NDF and ADF contents and rapid rumen degradation rates [23,24]. Its relative feeding value resembles silage maize, establishing it as an excellent unconventional roughage [25]. This study further demonstrated that including 60% garlic straw significantly enhanced dry matter digestibility, aligning with prior research [19,26].

### 4.2. Effects of Different Addition Ratios on VFA

VFAs are produced by rumen microbial fermentation [16,17]. However, there is no consensus on how garlic and its by-products influence rumen VFA. For instance, studies have shown that garlic oil had no significant effect on the total concentration of VFAs [18,19]. On the other hand, allicin has been reported to enhance total short-chain fatty acid (SCFA) engagement, organic matter (OM), and fiber digestibility in sheep. This increase is observed mainly in butyrate and propionate while simultaneously reducing acetate concentration. Notably, acetate is a pivotal substrate for methane production [20,21]. Therefore, by decreasing acetic acid production, it is possible to curtail methane emissions. Interestingly, our study revealed that adding silage garlic straw increased acetate and total VFA concentrations. While no significant disparity in the acetic acid concentration was observed among 0%, 20%, 80%, and 100% addition ratios, a notable difference emerged at 60% inclusion, marking the point at which the VFA concentrations peaked.

### 4.3. Effects of Different Addition Ratios on Microflora

Garlic straw, much like garlic itself, possesses antibacterial properties that inevitably influence the composition of rumen microbiota [22,23,24]. Rumen microorganisms play a pivotal role in rumen fermentation activity, directly impacting overall digestive efficiency [25]. Notably, *Campylobacter ales*, a common pathogenic strain associated with gastroenteritis and diarrhea in sheep, is particularly sensitive to these antibacterial effects [26]. Interestingly, we observed a significant reduction in the abundance of Campylobacter ales in direct correlation with increasing concentrations of garlic straw. Surprisingly, the broader microbial community remained largely resilient, even with modest additions (less than 20% concentration). This resilience could be attributed to the relatively low concentrations of antimicrobial compounds, potentially allowing for compensation in nitrogen metabolism, particularly in the production of NH3-N, which might mitigate the impact of garlic straw on the microbiota [27,28]. However, higher proportions of addition reduced VFAs, underscoring the antibacterial effect of garlic straws.

The rumen is a vital ruminant organ responsible for digesting roughage and concentrated feed. The microorganisms within the rumen work together to break down and ferment feed into volatile fatty acids, providing the energy necessary for growth and maintenance [29]. The rumen harbors various microorganisms, including functional groups involved in fiber degradation, protein breakdown, lipid metabolism, and carbohydrate digestion. The composition and abundance of these microorganisms influence rumen development in ruminant animals. Additives in the diet may alter the rumen’s design and types of microorganisms. The results of this experiment indicate that the alpha diversity in different treatment groups revealed that garlic straw with silage corn stalks did not change the diversity and abundance of rumen microorganisms in Hu sheep. Its impact on the microbial structure in the rumen was relatively limited.

In the rumen of ruminant animals, the dominant phyla are Firmicutes and Bacteroidetes, which aligns with the findings of this experiment. Firmicutes primarily degrade cellulose and can produce butyric acid, serving as a direct energy source for intestinal epithelial cells. Microbial fermentation transforms cellulose into short-chain fatty acids, providing approximately 10% of the body energy needs. Bacteroidetes play a crucial role in the fermentation process within the rumen of ruminant animals, particularly in the breakdown and absorption of non-fiber plant components, including polysaccharides, carbohydrates, fats, and proteins. They are the rumen’s primary degraders of non-fiber plant components [30]. In this study, the dominant genera in the G80 group were primarily *Ruminococcus* and *Prevotella*. *Ruminococcus*, a major fiber-degrading bacterium in the rumen, is one of the most abundant genera in the order Clostridiales under the phylum Firmicutes. It produces a significant amount of cellulase and hemicellulase, facilitating the degradation of dietary cellulose and producing volatile fatty acids [31]. *Prevotella*, conversely, is highly active in the breakdown of hemicellulose and plays a crucial role in the degradation of non-fiber polysaccharides and proteins. Additionally, *Prevotella* can digest and utilize starch, xylan, and pectin, generating short-chain fatty acids, including acetic acid, succinic acid, and propionic acid [32]. Interestingly, the G80 group exhibited a significant reduction in gas production compared to the other groups. We attribute this observation to the dominance of the Ruminococcus and Prevotella genera; however, further experiments are needed to substantiate this hypothesis. In addition, one of the drawbacks of this study is that it was conducted entirely in vitro.

## 5. Conclusions

The combination of garlic straw with silage corn stalks proves highly beneficial for feeding Hu sheep. It significantly enhances dry matter digestibility and in vitro gas production, with the most notable effects observed at a 60% inclusion rate when compared to the G0 group. Moreover, it positively influences Hu sheep’s rumen microbiota, particularly favoring the dominant bacterial groups, such as Prevotella, Rikenellaceae RC9 gut group, and Succiniclasticum. This finding provides valuable insights for mitigating greenhouse gas emissions from ruminant animals. It establishes a theoretical foundation for effectively utilizing garlic straw as a feed resource for Hu sheep and offers a recommended proportion for the optimal combination of garlic straw with silage corn stalks.

## Figures and Tables

**Table 1:** Nutritional composition of feed ingredients of the diet fed to Hu sheep (%, dry matter basis).

Ingredients	DM	CP	NDF	ADF	EE	Ca	P	RFV
Garlic straw	53.36	7.29	41.11	34.34	2.52	2.05	0.19	140.63
Silage corn stalks	32.29	6.96	58.52	32.98	1.27	0.45	0.20	143.03

Note: dry matter (DM); crude protein (CP); neutral detergent fiber (NDF); acid detergent fiber (ADF); ether extract (EE); relative feeding value (RFV). RFV = (120/NDF) × (88.9 − 0.779×ADF)/1.29.

**Table 2:** Composition and nutrition of ration (%, dry matter basis).

Ingredients	Recipe Contents	Nutritional Indicators	Nutritional Levels
Corn	43.00	Crude protein (CP)	17.06
Soybean meal	9.20	Crude fiber (CF)	5.52
Bran	10.00	Ether extract (EE)	3.80
Protein powder	7.00	Ash	10.95
Distiller-dried grains with solubles	11.48	Neutral detergent fiber (NDF)	19.24
Rice oil	4.96	Acid detergent fiber (ADF)	6.33
Germ meal	4.95	Ca	1.87
Peanut vine	4.96	P	0.67
Premix	4.00	/	/
Lysine	0.15	/	/
Methionine	0.05	/	/
Guanidinoacetic acid	0.04	/	/
Sodium chloride	0.21	/	/
Total	100.00	/	/

Note: The premix used in the test formula is the same as the premix used in the original feed formula of the sheep farm; nutritional levels were all measured values. The premix provides the following per kg measure of total mixed ration: VA 15,000 IU, VD 4000 IU, VE 50 mg, VK3 0.5 mg, VB1 1.4 mg, VB25 mg, VB63 mg, Fe 75 mg, Cu 8 mg, Mn 90 mg, Zn 80 mg, Se 0.3 mg, I 0.8 mg.

**Table 3:** The artificial rumen fluid consists of the following components.

Items	Composition
Liquid A	Mix and dissolve CaCl_2_·2H_2_O 13.2 g, MnCl_2_·4H_2_O 10.0 g, CoCl_2_·6H_2_O 1.0 g, and FeCl_3_·6H_2_O 8.0 g in distilled water at constant volume to 100 mL
Solution B	Dissolve 4.0 g NH_4_CO_3_ and 35 g NaHCO_3_ in 1000 mL distilled water
Liquid C	Dissolve 5.7 g NaH_2_PO_4_, 6.2 g KH_2_PO_4_, and 0.6 g MgSO_4_·7H_2_O in distilled water at constant volume to 1000 mL
Azurazine solution	0.1% (*w*/*v*)
Reducing agent solution	Mix 95 mL distilled water, 4.0 mL 1N NaOH, and 0.625 g Na_2_S·9H_2_O

Note: all reagents were domestically analytically pure.

**Table 4:** Effects of garlic straw and silage corn stalks ratio on gas production, pH, and fermentation solution digestibility.

Item/Proportion	CON	G0	G20	G40	G60	G80	G100	SEM	*p*-Value		
									Total	Linear	Quadratic
Gas/mL	18.00 ^c^	49.25 ^a^	50.25 ^a^	50.00 ^a^	52.67 ^a^	38.67 ^b^	43.00 ^ab^	2.58	<0.001	0.207	0.441
pH	6.67 ^a^	6.46 ^b^	6.47 ^b^	6.51 ^b^	6.57 ^ab^	6.59 ^ab^	6.56 ^ab^	0.02	<0.001	0.022	0.313
IVDMD/%	100.00 ^c^	37.04 ^b^	21.07 ^ab^	44.20 ^b^	51.14 ^a^	41.93 ^a^	31.64 ^a^	0.05	<0.001	0.692	0.414

Note: In peer data, different lowercase letters of shoulder labels indicate a significant difference (*p* < 0.05), and the same or no letters indicates no significant difference (*p* > 0.05). CON: no dry matter, only digestive fluid containing rumen fluid. G0–G100 represents the ratio of the garlic straw/silage corn stalks (e.g., G20, garlic straw/silage corn stalks = 20:80).

**Table 5:** Effects of garlic straw with silage corn stalks on volatile fatty acids for in vitro rumen fermentation (% of total VFA).

Proportion (mmol/L)	CON	G0	G20	G40	G60	G80	G100	SEM	*p*-Value		
									Total	Linear	Quadratic
Acetic acid	16.24 ^c^	23.39 ^ab^	21.87 ^ab^	20.64 ^b^	25.30 ^a^	21.38 ^ab^	22.03 ^ab^	3.40	<0.001	0.853	0.996
Propionic acid	7.72 ^a^	11.10 ^c^	10.60 ^bc^	8.66 ^ab^	11.58 ^c^	9.62 ^abc^	8.31 ^ab^	1.94	<0.001	0.318	0.768
Butyric acid	4.72 ^b^	5.59 ^a^	5.17 ^ab^	4.82 ^b^	5.67 ^a^	4.85 ^b^	5.17 ^ab^	0.51	<0.001	0.575	0.669
Total VFA	28.68	40.08	37.64	34.12	42.55	35.85	35.51	/	/	/	/
Acetic acid/ Propionic acid	2.10	2.11	2.06	2.38	2.18	2.22	2.65	/	/	/	/

**Note:** In the provided data, distinct lowercase letters on the shoulder label signify a significant difference (*p* < 0.05. The same or no letters indicates no significant difference (*p* > 0.05).

## Data Availability

The data presented in this study are available on request from the corresponding author. The data are not publicly available due to the privacy or ethical restrictions.
